# The protective role of prebiotics and probiotics on diarrhea and gut damage in the rotavirus-infected piglets

**DOI:** 10.1186/s40104-024-01018-3

**Published:** 2024-05-03

**Authors:** Heng Yang, Xiangqi Fan, Xiangbing Mao, Bing Yu, Jun He, Hui Yan, Jianping Wang

**Affiliations:** Institute of Animal Nutrition, Sichuan Agricultural University, Key Laboratory for Animal Disease-Resistance Nutrition of China Ministry of Education, Key Laboratory of Animal Disease-Resistant Nutrition and Feed of China Ministry of Agriculture and Rural Affairs, Key Laboratory of Animal Disease-Resistant Nutrition of Sichuan Province, No. 211, Gongpinghuimin Road, Wenjiang District, Chengdu, Sichuan Province 611130 People’s Republic of China

**Keywords:** Diarrhea, Piglet, Prebiotics, Probiotics, Rotavirus

## Abstract

Rotavirus is one of the pathogenic causes that induce diarrhea in young animals, especially piglets, worldwide. However, nowadays, there is no specific drug available to treat the disease, and the related vaccines have no obvious efficiency in some countries. Via analyzing the pathogenesis of rotavirus, it inducing diarrhea is mainly due to disturb enteric nervous system, destroy gut mucosal integrity, induce intracellular electrolyte imbalance, and impair gut microbiota and immunity. Many studies have already proved that prebiotics and probiotics can mitigate the damage and diarrhea induced by rotavirus infection in hosts. Based on these, the current review summarizes and discusses the effects and mechanisms of prebiotics and probiotics on rotavirus-induced diarrhea in piglets. This information will highlight the basis for the swine production utilization of prebiotics and probiotics in the prevention or treatment of rotavirus infection in the future.

## Introduction

Severe diarrhea is the primary cause of dehydration in the acute gastroenteritis, which can lead to some complications (even death) if leaving it untreated. Many viruses can induce acute gastroenteritis, such as rotavirus, norovirus, adenovirus, astrovirus, and calicivirus. Rotavirus is one of the main pathogens causing diarrhea in young animals (i.e., piglets). It mainly infects host duodenal and jejunal mucosal epithelial cells through the fecal–oral route, which will induce cell apoptosis and villi atrophy in gut, and then lead to malabsorption and diarrhea [1]. Piglets are extremely susceptible to rotavirus. During 1–4 weeks of age, the comprehensive infection rate is as high as 80%, and the mortality rate can reach up to 20% in piglets [2]. It has seriously impaired the health and growth of pigs all over the world, especially developing countries [3, 4].

Probiotics that are derived from Latin mean “for life”. Before probiotic microbes are discovered and utilized, their fermented products (such as beer, bread, wine, yogurt, mare’s milk wine, and cheese) have used for nutritional and therapeutic purposes [5]. Probiotics have a variety of beneficial effects on the body, including anti-inflammation, anti-oxidation, anti-cancer, anti-virus, and regulating intestinal flora and immune function [6–12]. Recently, prebiotics are developed as one of antibiotic substitutes [13]. This is mainly due that prebiotics may act as substrates for probiotics [14]. Moreover, the combination of probiotics and prebiotics as synbiotics can also regulate health of hosts [15]. Therefore, many studies have already focused on the curing and preventing effect of probiotics, prebiotics and synbiotics on the rotavirus-induced diarrhea via protecting gut health in piglets and some related models.

## Rotavirus

Rotavirus is a member in the genus *Rotavirus* in the family Reoviridae, and its complete three-layered particles (inner, middle, and outer capsid layers) resemble a wheel with a smooth surface under electron microscopy [16]. The rotavirus genome is composed of 11 segments of double-stranded RNA encoding viral proteins (VP1–VP4, VP6 and VP7), and nonstructural proteins (NSP1–NSP5/6) [17–20]. According to the antigenicity of their VP6 protein, rotavirus are classified into ten different groups (A–J), whose A, B, C, E and H can only infect pigs; and rotavirus A is the highest prevalence, and one of the most obvious reasons of acute diarrhea of piglets [16].

## Rotavirus pathogenesis

After rotavirus infects a juvenile host, it replicates primarily in epithelial cells of the middle and apex of the small intestine, inducing villi blunting and atrophy, thereby further impairing the intestinal absorptive surface area and hindering the absorption of essential nutrients such as carbohydrates, proteins, and fats [16]. In addition, right ventricular infection can lead to electrolyte imbalances and gut microbiome imbalances, which can further exacerbate intestinal damage. The combination of these factors leads to the occurrence of clinical symptoms associated with rotavirus infection (such as diarrhea, vomiting, fever, anorexia, and weight loss) [20]. In addition, some evidences have shown that rotavirus pathology can be systemic [21]. Rotavirus inducing diarrhea may have some mechanisms, as follows.

### Rotavirus disturbs enteric nervous system

The intestinal motility is mainly regulated by the enteric nervous system, and 60% of cluster cells in the small intestine and 40% of cluster cells in the large intestine are in contact with nerve fibers [22]. Rotavirus infection down-regulates tyrosine hydroxylase in norepinephrine-sympathetic neurons, thereby reducing dopamine production [23]. This decreases norepinephrine production, and ultimately increases peristalsis in small intestine [23]. Rotavirus also activates intestinal glial cells through serotonin released by enterochromaffin cells, and then increases intestinal peristalsis [24]. These two effects can synergistically induce rapid intestinal peristalsis, causing host abdominal pain and diarrhea. Besides, rotavirus can activate vagus nerve afferents, and project into the medullary vomiting center of central nervous system, resulting to solitary nucleus excitation in the brainstem that dominates vomiting and diarrhea, and eventually form vomiting and diarrhea reflexes [25, 26]. Thus, rotavirus-induced diarrhea and some other symptoms are associated with abnormal stimulation of the enteric nervous system.

### Rotavirus destroys gut mucosal integrity

Gut mucosa plays an important role in nutrient digestion and absorption, and barrier function. Rotavirus infection causes the damage of host intestinal mucosa (especially villi) through affecting the survival of intestinal epithelial cells [27]. Our studies have also shown that rotavirus could induce apoptosis and autophagy in the jejunum of piglets [28–33]. On the one hand, this leads to a deficiency in nutrient absorption, and water-electrolyte disturbances, which will induce subsequent malabsorptive diarrhea [34, 35]. On the other hand, rotavirus doing harm to gut mucosa will impair the expression and generation of tight-junction proteins (including zonula occludens 1, occludin and claudins) and mucins [28, 29, 31, 33, 36], which could induce the diarrhea via the secondary infection of other pathogens. In this process, it mainly exists three mechanisms, as follows.

#### Rotavirus induces oxidative stress

Redox balance is critical for the survival of cells (i.e., intestinal epithelial cells). Our recent studies in piglets found that rotavirus infection enhanced the level of reactive oxygen species in small intestine (data not published), and also showed that rotavirus challenge inhibited total antioxidant capacity and total superoxide dismutase activity, and increased malondialdehyde level in the small intestine of piglets [29, 31]. In the mouse model, Sodhi et al. [37] also got the similar results. In the rotavirus-infected IPEC-J2 cells, the mRNA levels of AMP-dependent protein kinase (*AMPK*), total nuclear factor erythroid 2-related factor 2 (*Nrf2*) and its downstream factors (including manganese superoxide dismutase, catalase, glutathione peroxidase, heme oxygenase 1 and quinone oxidoreductase 1) were down-regulated [36]. These studies proposed that rotavirus should impair intestinal mucosal integrity via disturbing antioxidant capacity, which was involved into AMPK-Nrf2 signaling pathway.

Besides, Cleary and Ashkenazi found that excess of peroxides and free radicals could stimulate intestinal glands, promote gland secretion, and induce host diarrhea, which indirectly illustrated that rotavirus impairing antioxidant capacity was also a potential mechanism of diarrhea [38].

#### Rotavirus stimulates some apoptosis- and autophagy-related pathways

In the epithelial cells of host small intestine, rotavirus also damages the intestinal epithelial mucosa by affecting autophagy- and apoptosis-related pathways. (1) Calcium/calmodulin dependent kinase 2 (CAMK2) and Ca^2+^-related adenosine 5´-monophosphate (AMP)-activated protein kinase (AMPK) pathways. The NSP4 protein of rotavirus can induce the release of endoplasmic reticulum calcium into the cytoplasm of infected cells, thereby activating CAMK2 and the Ca^2+^-related AMPK pathways to trigger autophagy, thereby facilitating rotavirus replication. Subsequently, rotavirus replication and maturation require the transfer of NSP4 to a new endoplasmic reticulum (ER) [39]. (2) LC3II-related pathway. The newly formed NSP4 and VP7 fuse with COPII vesicles and exit ER, then interact with the autophagy protein LC3II to induce cell autophagy and release new viruses [40]. (3) Protein kinase R-like endoplasmic reticulum kinase (PERK)/eukaryotic initiation factor 2α (eIF2α) pathway. Rotavirus infection induces endoplasmic reticulum stress, thereby increasing PERK activity, leading to eIF2α phosphorylation, which selectively activates cAMP-dependent transcription factor 4 translation, and promotes of C/EBP homologous protein expression, and then inhibits B-cell lymphoma 2 expression, and induces of B-cell lymphoma 2-associated X transport from cytoplasm to mitochondria, thereby initiates cell apoptosis [30, 41, 42].

#### Rotavirus impairs endocrine in gut

The intestinal endocrine hormones play a vital role in the proliferation and survival of epithelial cells. Glucagon like peptide-2 (GLP-2), a 33-amino acid proglucagon-derived peptide, is generated and secreted by enteroendocrine cells of gut, which can promote intestinal growth and mucosal repair, increase epithelial cell proliferation, and inhibit enterocyte apoptosis [43]. Our previous study found that rotavirus challenge reduced GLP-2 level in the jejunal mucosa of piglets [29]. Thus, rotavirus destroying gut mucosa integrity is associated with the negative effect on gut endocrine.

### Nonstructural protein 4 induces intracellular electrolyte imbalance

NSP4, a non-structural protein of rotavirus, is defined as a viral enterotoxin [44]. Except for impairing gut mucosal integrity, NSP4 may induce diarrhea by impairing intracellular electrolyte balance. (1) Via binding cell receptors, NSP4 boosts the production of 1,4,5-triphosphate-regulated inositol, and further activates the phosphatidyl inositol signaling pathway, which mobilizes extracellular Ca^2+^ inflow, in turn stimulates chloride (Cl^−^) secretion pathways to increase the release of Cl^−^ into the lumen [45, 46]. (2) NSP4 synthesis inhibits the activity of the sodium (Na^+^) glucose co-transporter SGLT1 by activating TMEM16A channel, thereby inhibiting the intracellular transport of Na^+^ [47, 48]. (3) NSP4 can also activate the release of extracellular adenosine 5´-bisphosphate (ADP) of P2Y1 purinergic receptors on neighboring cells, which produces paracrine signals that may be manifested as intercellular Ca^2+^ waves, further amplifying electrolyte disorder of host cells and changing gastrointestinal physiology [49]. Intracellular electrolyte imbalance will lead to the decreased membrane stability and intracellular water loss, ultimately inducing diarrhea [50].

### Rotavirus affects host immunity

As a pathogen, rotavirus can invade hosts (especially intestine), but not be cleaned by the anti-viral immunity. This is derived from some rotavirus-resistant mechanisms.

#### To affect the immune-related cells

Some studies have shown that rotavirus infection can interfere the role of immune-related cells in animals. (1) Dendritic cells. In mice, rotavirus infection increased the expression of major histocompatibility complex II, CD40, CD80, and CD86 in mesenteric lymph nodes dendritic cells, which synergistically regulate the maturation, differentiation, and function of dendritic cells. Dendritic cells are divided into CD11b^+^ and CD11b^−^ dendritic cells. CD11b^+^ dendritic cells promote antigen presentation, inflammatory response and regulating T cell activation. CD11b^−^ dendritic cells play an important role in antigen capture and processing, and immune regulation [51–53]. Rotavirus infection affecting those protein expression would increase the percentage of CD11c^+^CD11b^+^CD8a^−^ dendritic cells in the mesenteric lymph nodes and decrease the proportion of CD11c^+^CD11b^−^CD8a^+^ dendritic cells, thereby downregulating immune response, leading to worsening of infection [54]. (2) Natural killer (NK) cells. Comstock et al. [55] found that rotavirus infection can reduce the number of NK cells in the mesenteric lymph nodes and ileal Peyer’s nodes of piglets. Vitamin A deficiency in sows infected with rotavirus had the decreasing number of NK cells [56]. In addition, it was found that rotavirus infection promotes immature NK cell activation, leading to cytotoxicity of bile duct cells and causing bile duct obstruction in mice [57]. Therefore, rotavirus infection can impair the number and activity of NK cells, and NK cell function, thereby lowering host immunity or causing other diseases. And this is related to the nutritional status of hosts. (3) Some other cells. In newborn piglets, rotavirus infection can reduce the total number of CD14 cells and monocytes in the intestinal lymph nodes, and also decrease the macrophage population in the mesenteric lymph nodes and ileal Peyer’s nodes [55]. González et al. [58] found that rotavirus infection reduces the activation of monocytes, thereby reducing uptake/binding with rotavirus-like particles. In addition, it was found in the rotavirus-infected mouse model that rotavirus infection increased the mRNA expression levels of macrophage inflammatory protein-1 and monocyte chemotactic protein-1 in the duodenum, jejunum, and ileum tissues [59]. In summary, rotavirus infection can reduce the number and activity of CD14 cells, monocytes and macrophages in host, and stimulate the expression of pro-inflammatory factors/chemokines secreted by these cells, thereby causing a decrease in immune function, and inducing inflammation.

#### To impair the pattern recognition receptors

Some components of rotavirus are a pathogen-associated molecular patterns, and it can be recognized by pattern recognition receptors (PRRs) in intestinal epithelial cells or immune cells [60]. However, rotavirus has 2 ways to escape from the related immune response. (1) NSP1, a non-structural protein of rotavirus, may play a role for hiding viral RNA, which prevents PRRs recognizing the virus, and reduces antiviral response of interferon (IFN) [61]. (2) NSP1 and VP3 (known as a viral protein) induce PRRs (such as retinoic acid-induced gene I, mitochondrial antiviral-signaling protein) degradation and/or decrease their formation, and degrade interferon regulatory factors (IRF), including IRF3, IRF5, IRF7, and IRF9 [62–65]. This will block the generation of host IFNs and pro-inflammatory cytokines, and decrease interferon-stimulated factor 3 formation, and eventually reduce the host innate antiviral immune activity [64, 66–70].

#### To regulate some immune-related pathways

Rotavirus can also impair the host’s immune response by regulating some immune related pathways. (1) Rotavirus-infected MA104 cells avoids the nuclear accumulation of activated signaling and transcriptional activating protein 1/2, and then effectively blocks the expression of type I and type II IFN-induced genes, which in turn reduces endogenous *ISG* mRNA levels [71]. (2) NSP1 in MA104 cells can inhibit the activation of NF-κB pathway by degrading E3 ubiquitin ligase complex, and inhibit the generation of host IFNs and pro-inflammatory cytokines [67, 72, 73]. (3) Rotavirus-infected piglet intestine and IPEC-J2 cells can also up-regulate microRNA-155-5p expression, and further targets and inhibits the mRNA levels of *TBK1*,* IRF3* and *IFN-β*. This regulates TBK1/IRF3 signaling pathway [74].

#### To influence the immune factors

Adaptive immunity against rotavirus can be used for virus identification, clearance, and placement for reinfection [3]. In the early stage of rotavirus infection, a large amount of rotavirus-IgM antibodies are produced in host. However, these antibodies have few roles for virus neutralization, and are only used as a symptom of infection. After 5 d of rotavirus infection, the rotavirus-IgG antibodies will be generated by host, which are mainly used to neutralize virus. Our studies also found that rotavirus challenge could enhanced serum and/or intestinal rotavirus specific antibodies in piglets [28, 29, 33, 60, 70, 75]. Li et al. [76] reported that rotavirus infection increased the serum concentration of rotavirus specific IgM and IgG in piglets. In addition, Preidis et al. [77] got the similar results in mice.

Certainly, rotavirus infection also affects the innate immunity related factors. Zhao et al. [78] found that rotavirus infection increased serum levels of IL-2, IL-4 and IFN-β in weaned piglets, as well as increased the expression of interferon-stimulated gene 15 (ISG15). Chen et al. [75] reported that, compared with normal piglets, rotavirus-challenged piglets have the higher levels of serum IgA and IgG, and jejunal and ileal IL-2 levels. Moreover, in the rotavirus-infected mice, the generation of intestinal secreting immunoglobulin A, and serum interferon-γ (IFN-γ) and tumor necrosis factor-α was promoted [79].

However, some previous studies have shown that there are the suppression of antiviral immune response and cytokine generation in host during the early stage of rotavirus infection [80, 81]. Thus, the difference of these results in immune factors could be mainly derived from the infectious duration. And these also may explain why the symptom of rotavirus infection mainly occur in the early stage of infection, and gradually self-heal after 5 d of infection.

### Rotavirus impairs intestinal microbiota

Gut ecosystem depends on the diversity and integrity of intestinal microflora, which is indispensable for many physiological processes, and plays a vital role in health (especially gut health) [82]. In the process of rotavirus-induced gastroenteritis, microbiota diversity was temporarily reduced, and microbiota composition was significantly altered [83]. Zhao et al. [84] determined the colon and rectum digesta of mice with rotavirus infection, and found that the relative abundance of Proteobacteria and Candidatus was increased, and the relative abundance of Firmicutes, Fusobacteria, Bacteroidetes, and Actinobacteria was decreased. Our study also reported that oral gavage of rotavirus resulted in a decrease in the total number of bacteria, lactobacilli, and bifidobacteria, and an increase in the population of *Escherichia coli* in the ileum and cecum digesta of piglets [28, 29, 33]. These results indicate that rotavirus infection induces a decrease in the total number of bacteria and beneficial bacteria in the host’s gut, as well as an enhancement in the number of harmful bacteria, which lead to the dysbiosis of gut microbiota and the increasing diarrhea duration. Thus, the pathogenicity of rotavirus is closely involved into the change of intestinal microbiota.

## Probiotics and/or prebiotics alleviates the rotavirus-induced diarrhea

Due to the diversity and variability of rotavirus species, there is currently no specific drug available to treat rotavirus infection. In swine production, rotavirus can’t be eradicated. Most producers mainly use antibiotics to reduce mortality and diarrhea, and further minimize the impact of rotavirus infection on swine production [3, 4]. In addition, some vaccines to rotavirus have already researched and used [85–89]. However, because of the difference in age, sex, weight, environment, gut microbiome and genome, the application of these vaccines can’t achieve the expected results, especially in livestock [90]. Then, some dietary interventions ways (including nutrients, and functional materials) are used to prevent and cure the rotavirus-induced diarrhea [32, 60, 74, 91–93]. Probiotics, prebiotics and synbiotics have positive effects on gut health, which leads to their utilization of alleviating rotavirus-induced gastroenteritis in many studies of pigs and other animal models.

### Probiotics

Nowadays, probiotics are internationally recognized as the live and non-pathogenic microorganisms that provide health benefits to hosts when administered in sufficient amounts [94]. Probiotics are mainly used to improve microbial balance in the gastrointestinal tract [8], which mainly contain *Bifidobacterium*, *Lactobacillus* and some other bacteria. Besides these, their roles may be played via food (for example, dairy products) fermentation and drug development. As an alternative to antibiotics, they have now become a research hotspot in animal production. There are a plenty of studies about the protective effects of probiotics on rotavirus infection (Table 1).

**Table 1:** The effect of probiotics on rotavirus infection in the in vivo and in vitro studies

Probiotics	Dose and duration	Objects	Rotavirus treatment	Effects of probiotics	References
*Bifidobacterium bifidum*	Oral infusion; 3 × 10^8^ CFU daily; 28 d	Newborn BALB/c mice	On d 5, 10 mL mice rotavirus (EDIM 5099, TCID_50_ = 2 × 10^7^/mL)	Rotavirus infection was delayed, the duration of infection was shortened, and the content of rotavirus in digesta was reduced on d 2–10	[6]
*Bifidobacterium breve* (YIT4064)	Supplementing in diets; 0.05%; 9 weeks	5-week-old female BALB/c mice and their newborn pups	Rotavirus (SA-II, serotype 3); Female mice (10^6^ PFU), 9–12 d before delivery; 5-day-old pups (2 × 10^6^ PUF)	The intestinal immunity was improved, and various mucosal tissue infection was decreased	[95]
*Bifidobacterium bifidum* and *Bifidobacterium infantis*	Oral infusion; A mixture (0.75 × 10^8^ CFU/mL of *Bifidobacterium bifidum* + 0.75 × 10^8^ CFU/mL of *Bifidobacterium infantis*); 1–3 weeks, 10 μL daily, 4–5 weeks, 20 μL daily, 6–7 weeks, 40 μL daily	Newborn BALB/c mice	On d 5, 10 μL rhesus rotavirus (2 × 10^7^ PFU/mL)	The onset and early resolution of diarrhea were delayed. And the immunity was increased	[96]
*Bifidobacterium thermophilum* (RBL67)	Oral infusion; 1 × 10^9^ CFU daily, d 3–9	3-day-old CD-1 mice	On d 9, ape rotavirus (SA-11, 1 × 10^4^ PFU	The duration of diarrhea and viral replication in gut were decreased, epithelial lesions were limited, recovery was accelerated, and the humor immunity was stimulated	[97]
*Bifidobacterium bifidum* (G9-1)	Prophylactic administration: oral infusion, 3 × 10^9^ CFU daily, d 1–10 Therapeutic administration: oral infusion, 3 × 10^9^ CFU daily, d 1–10	2-day-old BALB/c mice	On d 3, rotavirus (SA11, 1.5 × 10^6^ PFU)	Both prophylactic and therapeutic administration relieved rotavirus-induced diarrhea. Therapeutic dosing also alleviated water uptake disorders caused by injury in the intestine and rotavirus infection, and reduced rotavirus titers in the mixture of cecal and fecal contents	[98]
*Bifidobacterium lactis* (HN019)	Oral infusion; 1 × 10^9^ CFU/piglet, d 1–8	3-week-old piglets	Natural infection	The severity of diarrhea was reduced, feed conversion was improved, and the immunity was increased in piglets	[99]
*Bifidobacterium longum* (subsp. Infantis CECT 7210)	Oral infusion; 10^9^ CFU daily, d 1–4	9-week-old BALB/c mice	On d 1, murine rotavirus (McN, 100 DD_50_)	Rotavirus shedding in mice feces was reduced	[100]
*Bifidobacterium longum* (SPM1205, SPM1206) or *Lactobacillus ruminis* (SPM0211)	Oral infusion; 1 × 10^9^ CFU/mL, 150–200 μL daily, d 6–8	7-day-old BALB/c mice	On d 1–5, 150–200 μL human rotavirus (KBPV-VR-47, 1.5 × 10^5^ PFU/mL)	Rotavirus infection was inhibited. And the immune function was improved	[101]
*Bifidobacterium breve* (M-16V), *Lactobacillus helveticus* (R0052), or *Lactobacillus salivarius* (PS2)	Oral infusion; 1 × 10^9^ CFU/100 g body weight daily, d 2–14 after birth	Newborn Lewis rats	On d 5, ape rotavirus (SA-11, 4 × 10^8^ TCID_50_/rat)	With the exception of *Lactobacillus salivarius* (PS2), all probiotics reduced diarrhea severity and morbidity. In addition, all *Lactobacillus* strains reduced viral clearance at one day after inoculation	[102]
*Lactobacillus rhamnosus* GG (ATCC 53103), and *Bifidobacterium animalis* (subsp. Lactis Bb12)	Oral infusion; d 3, *Bifidobacterium animalis* (subsp. Lactis Bb12), 1 × 10^5^ CFU; d 5, *Lactobacillus rhamnosus* GG (ATCC 53103) and *Bifidobacterium animalis* (subsp. Lactis Bb12), 1 × 10^5^ CFU	Newborn piglets	The vaccines for human rotavirus (G1P1A[8], 5 × 10^7^ FFU, d 6, 15 and 26) human rotavirus (G1P1A[8], 10^5^ FFU, d 27)	Rotavirus-induced diarrhea was decreased in pigs. The immune function was increased in the small intestine of pigs	[103–105]
*Lactobacillus rhamnosus* (GG)	Supplementing in diets; 10^9^ CFU/g diet; 20 d	21-day-old pigs	On d 15, 3 mL porcine rotavirus (OSU, 1.4 × 10^7^ TCID_50_/mL)	Rotavirus reproduction was inhibited, diarrhea was alleviated, and the jejunal mucosal barrier function was increased	[33]
*Lactobacillus rhamnosus* (GG)	Oral infusion; d 1, 10^3^ CFU, d 2, 10^4^ CFU, d 3, 10^5^ CFU, d 4, 10^6^ CFU, d 5, 10^7^ CFU, d 6, 10^8^ CFU, d 7, 10^9^ CFU, d 8, 10^10^ CFU, d 9, 10^11^ CFU, d 10–16, 10^12^ CFU	3-day-old gnotobiotic pigs	On d 10, human rotavirus (G1P1A[8], 10^5^ FFU)	The percentage of diarrhea, duration of diarrhea, and mean cumulative fecal scores were decreased. The ileal epithelial barrier function and the immune function of piglets were improved	[9]
*Lactobacillus rhamnosus* (GG)	Oral infusion; d 3–11 or 3–16, total 3.22 × 10^6^ CFU or 2.22 × 10^9^ CFU	Newborn gnotobiotic pigs	The vaccines for human rotavirus (G1P1A[8], 5 × 10^7^ FFU, d 5 and 15) Human rotavirus (10^5^ FFU), d 33	The development of immune system in the neonatal was improved	[106]
*Lactobacillus rhamnosus* (GG)	Oral infusion; d 3–11, 10^3^–10^6^ CFU, daily	Newborn gnotobiotic pigs	The trivalent rotavirus reassortant vaccine for human rotavirus (1.8 × 10^6^ PFU, d 5, 15 and 26) Human rotavirus (WA, 10^5^ FFU), d 28	Rotavirus-induced diarrhea and viral shedding was reduced by enhancing intestinal and systemic immune responses	[107]
*Lactobacillus rhamnosus* (GG)	1 × 10^6^ CFU/mL, 24 h	IPEC-J2 cell	Porcine rotavirus (OSU, MOI = 20, 1 h)	The innate immunity was regulated	[108]
*Lactobacillus rhamnosus* (GG)	Oral infusion; 1 × 10^6^ CFU, 1 × 10^8^ CFU or 1 × 10^10^ CFU daily; 8 d	Newborn Kunming mice	On d 4, 100 μL human rotavirus (G1P1A[8], 3 × 10^6^ PFU/mL)	The duration of diarrhea was shortened, and the jejunal vacuolation was reduced	[79]
*Lactobacillus rhamnosus* (GG)	Oral infusion; 10^6^ CFU daily; 7 d	3 to 4-week-old female BALB/c mice	On d 8, 200 μL porcine rotavirus (DN30209)	Weight gain reduction and intestinal villi shedding were ameliorated, and the immunity was regulated	[54]
*Lactobacillus rhamnosus* (GG)	Oral infusion; 1.5 × 10^8^ CFU/pup, 0.05 mL daily, 7 d	2-day-old Lewis rats	Simian rotavirus (SA-11, 1.4 × 10^8^ PFU/mL), 0.6 mL at d 3, and 1.2 mL at d 4	Rotavirus clearance in the body was increased, and colon swelling was reduced	[109]
Engineered *Lactobacillus rhamnosus* (GG) expressing IgG-binding domains of protein G	Oral infusion, d 1–4	4-day-old BALB/c mice	On d 1, rhesus rotavirus (10 μL)	The prevalence, severity, and duration of rotavirus-induced diarrhea was reduced	[110]
*Lactobacillus acidophilus* (NCFM™)	Oral infusion; 10^3^, 10^3^, 10^4^, 10^4^, 10^5^, 10^5^, 10^6^, 10^6^ and 10^6^ CFU, d 1–9	3-day-old gnotobiotic pigs	The vaccines for human rotavirus (G1P1A[8], 5 × 10^7^ FFU, d 3 and 13)	The production of CD8^+^ T cell responses in ileum and spleen was enhanced, and IgA and IgG in ileum and blood were increased	[111]
*Lactobacillus acidophilus* (NCFM™)	Oral infusion; 3.2 × 10^6^ CFU daily, d 1–9; 2.1 × 10^6^ CFU alternate-day, d 1–9	3-day-old gnotobiotic pigs	The vaccines for human rotavirus (G1P1A[8], 5 × 10^7^ FFU, d 6 and 16) Human rotavirus (10^5^ FFU), d 34	Rotavirus-induced diarrhea was reduced, and the protection of vaccine was improved	[112]
*Lactobacillus acidophillus* (NCDC 15)	Oral infusion; 1 × 10^9^ CFU daily, d 1–5	4-week-old calves	Natural infection	Rotavirus-induced diarrhea was relieved	[113]
*Lactobacillus reuteri* (DSM 17938, and ATCC PTA 6475)	Intragastric administration; 50 μL (2 × 10^9^ CFU/mL), d 1–10	4-day-old CD-1 mice	On d 3, rotavirus (ECwt, 1 × 10^3^ ID_50_)	Both probiotic strains shortened the duration of diarrhea, and improved intestinal histopathology, microbe and immunity	[77]
*Lactobacillus reuteri* (ATCC 23272) and Lactobacillus acidophilus (NCFM™)	Oral infusion; a mixture of 10^3^, 10^4^, 10^5^, and 10^6^ CFU (1:1) on d 3, 5, 7 and 9, respectively	Newborn gnotobiotic pigs	On d 4, human rotavirus (WA, 10^5^ ID_50_)	The immunity was improved	[114]
*Lactobacillus reuteri* (ATCC 23272) and *Lactobacillus acidophilus* (NCFM™)	Oral infusion; a mixture of 10^3^, 10^4^, 10^5^, 10^6^ and 10^6^ CFU (1:1) on d 3, 5, 7, 9 and 11, respectively	Newborn gnotobiotic pigs	On d 5, human rotavirus (WA, 10^5^ ID_50_)	The immunity was improved	[115, 116]
*Lactobacillus gasseri* (SBT2055)	Supplementing in the diet; 0.1%, 4 weeks before mating and continuing until 9 d after birth	Female BALB/c mice, and their pups	Simian rotavirus (SA-11); Dams: one week after mating, 0.5 mL 10^6.9^ TCID_50_/mL; pups: d 5 after birth, 0.05 mL 10^6.9^ TCID_50_/mL	The incidence of rotavirus-induced diarrhea in pups was reduced	[117]
*Lacticaseibacillus rhamnosus* (GG)	Oral infusion; 10^3^, 10^4^, 10^5^, 10^6^, 10^7^, 10^8^, 10^9^, 10^10^, 10^11^, and 10^12^ CFU, d 1–10	3-day-old gnotobiotic pigs	On d 9, human rotavirus (G1P1A, 10^5^ FFU)	Autophagy marker expression and apoptosis were reduced in rotavirus-infected piglets, and rotavirus-induced tissue damage was partially prevented	[118]
*Lacticaseibacillus rhamnosus* (GG)	Oral infusion; 10^3^, 10^4^, 10^5^, 10^6^ and 10^6^ CFU for d 1–5; 10^3^, 10^3^, 10^4^, 10^4^, 10^5^, 10^5^, 10^6^, 10^6^ and 10^6^ CFU, d 1–9	3-day-old gnotobiotic pigs	The vaccines for human rotavirus (G1P1A[8], 5 × 10^7^ FFU, d 3 and 13) human rotavirus (10^5^FFU), d 31	*Lacticaseibacillus rhamnosus* (GG) increased the protection of vaccine against diarrhea, enhanced the response of rotavirus-specific serum IgA antibodies, and increased the response of effector/memory T cells to the vaccine	[119]
*Lacticaseibacillus rhamnosus* (GG)	Oral infusion; 10^6^, 10^8^, and 10^10^ CFU, d 1–3	4-day-old BALB/c mice	On d 1, 10 μL rhesus rotavirus (2 × 10^7^ FFU)	*Lacticaseibacillus rhamnosus* (GG) reduced the prevalence, duration and severity of diarrhea. Combining with hyperimmune bovine colostrum (containing human rotavirus (WA, rotavirus3, rotavirus5 and ST3) antibodies), it further reduced diarrhea outcomes, prevented histopathological changes, and decreased enteroviral load	[120]
*Escherichia coli* (Nissle 1917)	Oral infusion; 2 mL of diluted human infant fecal microbiota stock, d 4; *Escherichia coli* (Nissle 1917) (1 × 10^9^ CFU), d 11	Newborn gnotobiotic pigs	On d 11, human rotavirus (WA, 1 × 10^6^ FFU)	*Escherichia coli* (Nissle 1917) reduced rotavirus fecal shedding and diarrhea in piglets	[121]
*Escherichia coli* (Nissle 1917)	Oral infusion; colonization commensal microbiota (10^5^ CFU), d 7; *Escherichia coli* (Nissle 1917) (10^5^ CFU) and ciprofloxacin (60 mg) daily, d 14–19	Newborn gnotobiotic pigs	On d 20, human rotavirus (WA, 2 × 10^6^ FFU)	*Escherichia coli* (Nissle 1917) increased the immunity of pigs, and relieved the effect of ciprofloxacin	[122]
*Escherichia coli* (Nissle 1917)	Oral infusion; human infant fecal microbiota, d 1–3; *Escherichia coli* (Nissle 1917) (1 × 10^5^ CFU) and tryptophan (0.4 g) daily, d 7–24	Newborn gnotobiotic pigs	On d 10, human rotavirus (WA, 1 × 10^6^ FFU)	There was the synergetic effect on rotavirus infection via regulating intestinal microbiota when *Escherichia coli* (Nissle 1917) was used with tryptophan	[123]
*Lactobacillus rhamnosus* (GG) and/or *Escherichia coli* (Nissle 1917)	Oral infusion; 10^5^ CFU, d 1	6-day-old gnotobiotic pigs	On d 14, human rotavirus (G1P[8], 1 × 10^6^ FFU)	*Escherichia coli* (Nissle 1917) reduced viral shedding titers, and improved humor immunity. *Escherichia coli* (Nissle 1917) had the best protective effect on human rotavirus, and stimulated the natural immune system and DC-IL-12-NK immune axis	[124, 125]
*Lacticaseibacillus rhamnosus* (GG), and *Escherichia coli* (Nissle 1917)	Oral infusion; 10^5^ CFU, d 1	3-day-old gnotobiotic pigs	The vaccines for human rotavirus (WAG1P, 1 × 10^7^ FFU, d 4 and 11; WA, 1 × 10^6^ FFU, d 18)	*Escherichia coli* (Nissle 1917) reduced rotavirus shedding and diarrhea severity. Both probiotics stimulated human rotavirus-specific IgA antibody titers in piglets, and *Escherichia coli* (Nissle 1917) also enhanced host immune function	[126]
*Lactococcus lactis* (subsp. Lactis JCM 5805) (heat-killed)	Oral infusion; 1 mg/mouse daily, d 1–10	5-day-old BALB/c mice	On d 3, rotavirus (50 μL, 3.3 × 10^7^ PFU/mL)	The retardation of body weight gain and fecal scores was improved, and rotavirus titer in the feces was reduced	[127]
*Pediococcus acidilactici*	In vitro: 1 × 10^5^ CFU/mL, 2 h In vivo: Oral infusion; 1, 4 and 7 d	IPEC-J2 cell 3-week-old specific pathogen-free mice	In vitro: porcine rotavirus (100 TCID_50_, 12 h) In vivo: on d 8–10, porcine rotavirus (100 TCID_50_/d)	In vitro: viral infection was reduced In vivo: the number of viral particles in the duodenum of mice was decreased	[128]
*Lacticaseibacillus rhamnosus* (R0011), *Bifidobacterium longum* (R0175), *Lactiplantibacillus plantarum* (299V), *Lacticaseibacillus paracasei* (A234), *Bifidobacterium lactis* (A026), and *Lactobacillus gasseri* (A237)	10^5^, 10^6^ or 10^7^ CFU/mL (3 mL), 16 h	IPEC-J2 cell	Porcine rotavirus (OSU, MOI = 26, 1 h)	Theses probiotics reduced the infection rate of rotavirus in IPEC-J2 cells, and the best effect is *Bifidobacterium longum* (R0175)	[129]
*Ligilactobacillus salivarius* (FFIG35 and FFIG58)	5 × 10^7^ cells/mL, 48 h	Porcine intestinal epithelial cell	Porcine rotavirus (OSU, MOI = 1, 16 h)	Rotavirus replication in cells was reduced via regulating immunity	[130]
Segmented filamentous bacteria	Oral infusion; Adult: 400 μL, at 6–8 week of age; Suckling: 50 μL, 2 and 7 d after birth	6 to 8-week-old C57BL/6 mice and their pups	Mice rotavirus (EC, 10^4^–10^5^ SD_50_)	Segmented filamentous bacteria were sufficient to protect mice from rotavirus infection and associated diarrhea, and induced host gene expression and epithelial cell renewal in ileum	[131]
Segmented filamentous bacteria	Oral infusion; 40 mg/mL, 200 μL, 7 d	4-day-old C57BL/6 mice, and B6. 129P2-Nos2tm1Lau/J(*nos2*^−^^/^^−^) mice	On d 8, mice rotavirus (EC, 10^5 ^SD_50_)	Segmented filamentous bacteria acted as a protective effect on rotavirus-infected mice, which was associated with the decreasing migration of intestinal cells	[132]

#### Bifidobacterium

*Bifidobacterium* belongs to the phylum Actinomycetes, and is one of the first microorganisms to colonize the intestines of neonatal animals [133]. This bacterium has beneficial effects on hosts, such as preventing diarrhea, building the healthy microbiome, regulating the immune system, improving lactose intolerance, lowering cholesterol, and preventing cancer [134, 135]. In many studies of mouse and rat model, oral administration of different Bifidobacteria (including *Bifidobacterium breve* (YIT4064 and M-16V), *Bifidobacterium bifidum*, *Bifidobacterium infantis*, *Bifidobacterium longum* (subsp. Infantis CECT 7210, SPM1205 and SPM1206), *Bifidobacterium animalis* (subsp. Lactis Bb12), *Bifidobacterium lactis* (HN019), and *Bifidobacterium thermophilus* (RBL67)) may inhibit rotavirus infection and rotavirus-induced diarrhea, which is embodied on the decrease of diarrhea severity, incidence, and duration, and the shortened recovery period for diarrhea [95–105]. And dietary *Bifidobacterium breve* YIT4064 supplementation for female mice can efficiently protect their pups against rotavirus infection [95]. Rigo-Adrover et al. [136] fermented formula with *Bifidobacterium breve* and *Streptococcus thermophilus*, and this milk could alleviate the effect of rotavirus challenge on suckling rats.

#### Lactobacillus

The genus *Lactobacillus* belongs to the group of lactic acid bacteria (LAB). Lactobacilli are Gram-positive, catalase-negative, non-spore forming, rod-shaped, and facultative anaerobic, which mainly produce lactic acid by fermentation [137, 138]. Lactobacilli are widely found in food, feed, plants, animals, and humans, where fermentation may happen [139]. Generally, *Lactobacillus* is considered as one of the most abundant microorganisms in the intestine of hosts, and is closely linked to gut health [140]. Many studies in pigs, calves and mice have shown that the administration of lactobacilli (including *Lactobacillus rhamnosus* GG (LGG), *Lactobacillus reuteri*, *Lactobacillus acidophilus*, and *Lactobacillus gasseri*) may improve growth performance, and alleviate the rotavirus-induced diarrhea [9, 33, 54, 77, 109, 110, 113–116], and then could reduce the rotavirus-induced diarrhea in their pups [117]. And even, the products (i.e., exopolysaccharide) of lactobacilli had the positive effect on improving rotavirus-induced diarrhea and viral shedding, and prevented the disruption of intestinal epithelial integrity [141].

In addition, the administration of lactobacilli (such as *Lactobacillus casei*, *Lactobacillus plantarum*) relieves the infection and harm of some viruses (including transmissible gastroenteritis virus, porcine epidemic diarrhea virus) in the gut of hosts [142, 143].

#### *Escherichia coli* Nissle 1917

*Escherichia coli* Nissle 1917 (EcN) is a Gram-negative probiotic. EcN has the antagonistic effects on a variety of intestinal pathogens, and regulates the secretion of immune factors, which can enhance immunity in hosts [144]. Oral gavage of EcN attenuated the negative effect of rotavirus infection on gut functions and immunity, and reduced the mean peak titer of viral shedding and diarrhea in piglets [121, 122, 124–126]. And there was the synergetic effect on rotavirus infection when EcN was used with other materials (such as tryptophan) [123].

#### Some other bacteria

Except for *Bifidobacterium* and *Lactobacillus*, some other probiotics have also displayed the protective role for rotavirus infecting hosts. (1) *Lacticaseibacillus rhamnosus* GG. Via oral infusion, it might relieve the rotavirus infection, diarrhea, and gut damage, and improve immune function, cell autophagy and apoptosis in piglets and mice [118–120]. (2) *Lactococcus lactis* (subsp. Lactis JCM 5805). Oral administration of heat-killed *Lactococcus lactis* (subsp. Lactis JCM 5805) improved the retardation of body weight gain and fecal scores, and reduced rotavirus titer in the feces of mice [127]. (3) *Pediococcus acidilactici*. Oral gavage of *Pediococcus acidilactici* decreased the number of viral particles in the duodenum of specific pathogen-free mice [128]. (4) *Lactiplantibacillus plantarum* (299V). Leblanc et al. [129] found that the incubation of *Lactiplantibacillus plantarum* (299V) reduced the infection rate of rotavirus in IPEC-J2 cells. (5) *Ligilactobacillus salivarius* (FFIG35 and FFIG58). Indo et al. [130] reported that the incubation of *Ligilactobacillus salivarius* (FFIG35 and FFIG58) reduced the rotavirus replication in the porcine intestinal epithelial cells. (6) Segmented filamentous bacteria (SFB). SFB are some Gram-positive clostridial bacteria, mainly colonizes the surface of epithelial cells at the end of ileum, and plays an important role in preventing the pathogenic microbial infection, and the occurrence and development of autoimmune diseases [145, 146]. Shi et al. [131] and Ngo et al. [132] reported that oral infusion of SFB alleviated the damage of intestines, and inhibited the viral replication and infection in the rotavirus-challenge mice.

### Prebiotics

In December 2016, the International Scientific Association for Probiotics and Prebiotics updated the definition of prebiotics as a class of substances that are selectively utilized by host microorganisms to confer health benefits on the host. Its broad definition may be involved into all substances that retain selectively microbiota-mediated mechanisms and must be beneficial for health [14]. At present, the main prebiotics include functional oligosaccharides, polysaccharides and natural plant extracts. The main purpose of prebiotics application is to stimulate the growth and activity of beneficial bacteria in the gastrointestinal tract, and has positive effects on host health via acting as a barrier to pathogens [13], and improving host immunity, antioxidant capacity and intestinal barrier [147].

Prebiotics also show the role for preventing rotavirus infection (Table 2). The studies of our lab have shown that supplementing pectic oligosaccharide, lentinan, or lactose in diets can efficiently improve intestinal microflora, gut barrier function and immunity, reduce diarrhea, and increase growth performance in the rotavirus-infected piglets [28, 29, 31, 70, 75]. Oral administration of β-glucan improved the health condition and reduced the piglet mortality from rotavirus associated diarrhea [148]. Supplementing human milk oligosaccharides or a mixture of short-chain galacto-oligosaccharides and long-chain fructo-oligosaccharides in formula reduced the duration of rotavirus-induced diarrhea in piglets partially by modulating colonic microbiota and immune response to rotavirus infection; and the effect of human milk oligosaccharides was better than that of the mixture [55, 76]. In the sows vaccinated with rotavirus, supplementing galacto-oligosaccharide in diets could reduce rotavirus infection in the neonatal piglets [149]; and oral administration of galacto-oligosaccharides reduced the incidence and severity of rotavirus-associated diarrhea in Lewis rats [150]. Rigo-Adrover et al. [136] reported that supplementing a mixture of short-chain galacto-oligosaccharides, long-chain fructo-oligosaccharides and pectin-derived acidic oligosaccharides in formula protected suckling rats from rotavirus gastroenteritis, and supplementing 2´-fucosyllactose and/or a mixture of short-chain galacto-oligosaccharides/long-chain fructo-oligosaccharides in formula reduced the clinical variables of rotavirus-induced diarrhea in newborn rats [151–153]. The treatment of polysaccharides from *Portulaca oleracea* L. could also reduce the expression of VP6, and viral titers during the internalization and replication phases of rotavirus in IPEC-J2 cells [154].

**Table 2:** The effect of prebiotics on rotavirus infection in the in vivo and in vitro studies

Prebiotics	Dose and duration	Objects	Rotavirus treatment	Effects of prebiotics	References
Human milk oligosaccharides, or a mixture (short-chain galacto-oligosaccharides and long-chain fructo-oligosaccharides (9:1))	Supplementing in formula; 4 g/L, 14 d	Newborn pigs	On d 10, 1 mL porcine rotavirus strain OSU (P9[7], g5, 5 × 10^6^ FFU)	Human milk oligosaccharides or the mixture shortened the duration of rotavirus-induced diarrhea, and modulated gut microflora and immune response	[55, 76]
A mixture of short-chain galacto-oligosaccharides, long-chain fructo-oligosaccharides, and pectin-derived acidic oligosaccharides (76.5: 8.5: 15)	Supplementing in formula; 0.8 g/100 g body weight daily, 14 or 21 d	3-day-old Lewis rats	On d 5, simian rotavirus (SA-11, 2 × 10^8^ TCID_50_/rat)	The mixture improved diarrhea and immunity in rotavirus-infected mice	[136]
2'-fucosyllactose, a mixture of short-chain galacto-oligosaccharides and long-chain fructo-oligosaccharides (9:1), and their combination	Supplementing in formula; 2´-fucosyllactose, 0.2 g/100 g body weight daily; a mixture of short-chain galacto-oligosaccharides and long-chain fructo-oligosaccharides, 0.8 g/100 g body weight daily; 7 d	Newborn Lewis rats	On d 5, simian rotavirus (SA-11, 4 × 10^8^ TCID_50_/rat)	All interventions showed reduction in clinical variables of rotavirus-induced diarrhea, and could promote the intestinal maturation and immune responses	[151, 153]
2'-Fucosyllactose	Supplementing in formula; 0.2 g/100 g body weight daily, 8 d	Newborn Lewis rats	On d 5, simian rotavirus (SA-11, 4 × 10^8^ TCID_50_/rat)	2'-Fucosyllactose alleviated diarrhea in rotavirus-infected rats	[152]
Lactose	Supplementing in diets; 4% or 6%, 20 d	21-day-old weaned pigs	On d 15, 5 mL porcine rotavirus (OSU, 10^6^ TCID_50_/mL)	Lactose alleviated the negative effects of rotavirus on growth, diarrhea, nutrient utilization, intestinal barrier function and immunity improvement	[28]
Galacto-oligosaccharides	Supplementing in diets; 30 g/d, 7 d before farrowing	Sows	The vaccines for porcine rotavirus (OSU 6, 2 weeks prior to farrowing) rotavirus infection in farms	Galacto-oligosaccharides increased rotavirus specific IgG and IgA in sow colostrum, and thereby reduced neonatal rotavirus infection	[149]
Galacto-oligosaccharides	Oral infusion; 0.8 g/100 g of body weight daily, 28 d	Lewis rats	On d 6, simian rotavirus (SA11, 2 × 10^8^ TCID_50_) On d 17, mice rotavirus (EDIM, 1.3 × 10^8^ TCID_50_)	Galacto-oligosaccharides reduced the incidence and severity of rotavirus-associated diarrhea	[150]
Lentinan	Supplementing in diets; 84 mg/kg, 19 d	21-day-old weaned pigs	On d 15, 4 mL porcine rotavirus (OSU, 10^6^ TCID_50_/mL)	Lentinan reduced rotavirus-induced diarrhea, and also decreased the effects of rotavirus on gut health	[31]
Lentinan	In vivo: supplementing in diets; 84 mg/kg, 19 d In vitro: 24 mg/L, 20 h before rotavirus infection and 3 d after rotavirus infection	21-day-old weaned pigs IPEC-J2 cell	In vivo: On d 15, 4 mL porcine rotavirus (OSU, 10^6^ TCID_50_/mL) In vitro: porcine rotavirus (OSU, MOI = 3), 1 h	Lentinan alleviated the effects of rotavirus on growth performance, diarrhea and intestinal immunity	[70]
Polysaccharides from *Portulaca oleracea* L	Before rotavirus infection, 400 μg/mL 6 h; After rotavirus infection, 400 μg/mL 24 h	IPEC-J2 cell	Porcine rotavirus ( MOI = 2), 1 h	The polysaccharides reduced the expression of VP6, and viral titers in IPEC-J2 cells	[154]
β-glucan	Oral infusion; 50 mg/kg body weight daily, 5 d	Piglets (0–2 weeks)	Natural infection	β-glucan improved the health condition and reduced the piglet mortality from rotavirus associated diarrhea	[148]
Stevioside, and/or sophora flavescens extract	Oral infusion; 4, 8 and 12 g stevioside, 400, 800 and 1,600 mg sophora flavescens extract, 4 times daily, d 2–8	3-day-old gnotobiotic pigs	On day 1, 3 mL porcine rotavirus (G5P[7], 5 × 10^5^ PFU/mL)	Stevioside had no effect on enteritis, but sophora flavescens extract partially improved enteritis. And the combination of stevioside and sophora flavescens extract reduced diarrhea, and improved small bowel lesion scores and fecal virus excretion	[155]
Resveratrol	Supplementing in diets; 3, 10 and 30 mg/kg, 21 d	28-day-old pigs	On d 22, 4 mL porcine rotavirus (AV59, 1 × 10^6^ TCID_50_/mL)	Resveratrol relieved diarrhea caused by rotavirus infection, reduced inflammation, and maintained immune function	[156]
Resveratrol	Oral infusion; 10 and 20 mg/kg body weight, d 1–5	3–5-day-old BALB/c mice	On d 1, 30 μL simian rotavirus (SA-11)	Resveratrol significantly reduced the severity of diarrhea, reduced viral titers, and alleviated the associated symptoms in rotavirus-infected mice	[59]
Resveratrol dimer trans-ε-viniferin	Oral infusion; 2.27 μg, three times daily, d 2–4	4–7-day- old C57BL/6 mice	On d 1, 25 μL, simian rotavirus (SA-11, 1.2 × 10^7^ PFU/mL)	Resveratrol dimer *trans*-ε-viniferin inhibited rotavirus-induced diarrhea in mice	[157]
Quercetin	Oral infusion; 10 mg/kg body weight, d 1–2 after virus inoculation	5-day-old BALB/c mice	On d 5, human rotavirus (EW, 10^7^ PFU) and simian rotavirus (SA11, 10^7^ PFU)	Quercetin decreased the expression of viral proteins and viral titers in the small intestine of rotavirus-infected mice	[158]
Baicalin	Oral infusion; 0.075, 0.15 and 0.3 mg/g body weight, d 1–3	4-day-old Kunming mice	On d 1, 200 μL, simian rotavirus (SA-11, 10^4.64^ TCID_50_/mL)	Baicalin can alleviate weight loss and reduce the incidence of diarrhea in the rotavirus-infected mice	[159]
Genipin	Oral infusion; 100 μmol/L, d 1–5	Newborn BALB/c mice	On d 5, 10 μL, mice rotavirus (EDIM, 2 × 10^7^ FFU)	Genipin shortened diarrhea duration and fecal viral shedding, as well as a reduction in damage to the intestinal epithelium of rotavirus-infected mice	[160]
Ziyuglycoside II	Oral infusion; 3, 6 and 9 μmol/L, d 1–7	3-day-old BALB/c mice	On d 1, 25 μL, simian rotavirus (SA-11)	The administration of Ziyuglycoside II improved diarrhea symptoms, and reduced diarrhea scores in the rotavirus-infected mice	[161]
Loureirin C extracted from *Dracaena cochinchinensis* S.C. Chen	Oral infusion; 2.72 μg, d 1–3	3-day-old ICR mice	On d 1, 25 μL simian rotavirus (SA-11, 1.2 × 10^7^ PFU/mL)	Loureirin C reduced fecal water content, intestinal motility rate, and smooth muscle contraction in rotavirus-infected mice	[162]
Ginsenoside-Rb2 and 20(S)-Ginsenoside-Rg3 from Korean Red Ginseng	Oral infusion; 75 mg/kg body weight, d 7–9	Newborn BALB/c mice	On d 10, simian rotavirus (SA-11, 1.5 × 10^6^ to 1.5 × 10^7^ PFU/mouse)	Ginsenoside reduced viral titers in the gut of rotavirus-infected mice	[163]
Deoxyshikonin	Intraperitoneally injecting, 5 and 10 mg/kg body weight, d 2	4-day-old BALB/c mice	On d 1, human rotavirus (WA) and simian rotavirus (SA-11)	Deoxyshikonin had anti-rotavirus function, increased survival and body weight, reduced diarrhea score in the rotavirus-infected mice	[164]
*Valeriana jatamansi* Jones extracts	Oral infusion; 10, 20 and 30 mg/kg body weight, d 1–14	3-day-old BALB/c mice	On d 1, 20μL, simian rotavirus (SA-11)	*Valeriana jatamansi* Jones extracts inhibited rotavirus-induced diarrhea in mice	[165]
Red wine extract	Oral infusion; 20 μL, twice daily, d 1–3	Newborn C57/BL6 mice	On d 1, 30 μL, simian rotavirus (SA-11, 1.2 × 10^7^ PFU/mL)	Red wine extract prevents rotavirus-induced diarrhea by inhibiting intestinal fluid secretion in mice	[166]
*Glycyrrhiza uralensis* extract	Supplementing in artificial milk; 100, 200 and 400 mg/mL, d 5–11	Newborn pigs	On d 3, 3 mL, porcine rotavirus (K85, 5 × 10^5^ FFU/mL)	*Glycyrrhiza uralensis* extract cured rotavirus-induced diarrhea, and improved small bowel lesion scores and fecal viral shedding in pigs	[167]
Saponin extracted from *Quillaja saponaria* Molina	Oral infusion; 0–0.5 mg/mouse, d 1–7	Newborn BALB/c mice	On d 3–7, rhesus rotavirus (ATCC VR-954, 50, 500, 5,000 and 50,000 FFU, respectively)	Saponin relieved the severity and duration of diarrhea caused by rotavirus in mice	[168]
Qiwei Baizhu powder	Oral infusion; 150 mL/kg body weight, d 3–5	Newborn NIH mice	On d 2, 50 μL, Human rotavirus (WA, 4 × 10^8^ PFU/mL)	Qiwei Baizhu powder is effective to rotavirus-induced gastroenteritis in neonatal NIH mice	[169]
Pectic oligosaccharide	Supplementing in diets; 200 mg/kg, 18 d	21-day-old weaned pigs	On d 15, 4 mL porcine rotavirus (OSU, 10^6^ TCID_50_/mL)	Pectic oligosaccharide improved the growth performance, diarrhea severity, gut health, immune function and nutrient utilization of rotavirus-challenged pigs	[29, 75]
*Macleaya cordata* extracts	Oral infusion; 1, 2 and 4 mg/kg body weight, d 1–7	3-day-old BALB/c mice	On d 3, 30 µL, simian rotavirus (SA-11, 4 × 10^7^ FFU/mL)	*Macleaya cordata* extracts were effective to improve rotavirus-induced diarrhea in a dose-dependent manner by decreasing viral RNA levels	[170]

Additionally, in piglets and some models with rotavirus infection, the administration of many plant extracts could relieve diarrhea, and improved small intestinal lesion scores and fecal virus excretion. These plant extracts are involved into resveratrol, ginsenoside, saponin, quercetin, loureirin, baicalin, ziyuglycoside II, genipin, deoxyshikonin, steviol glycosides, bitter ginseng extract, red wine extract, *Glycyrrhiza uralensis* extract, Qiwei Baizhu powder, *Macleaya cordata* extracts, and *Valeriana jatamansi* Jones extracts [59, 155–170].

### Synbiotics

Synbiotics are defined as “mixtures of probiotics and prebiotics that beneficially affects the host by improving the survival and implantation of live microbial dietary supplements” [171]. Synbiotics have the characteristics of probiotic and prebiotic, and were produced for overcoming some possible difficulties of probiotics survival in the gastrointestinal tract [172]. Therefore, an appropriate combination of both components should ensure a superior role, compared to the activity of probiotics or prebiotics alone [173]. From now on, there are several studies about the effect of synbiotics on the rotavirus-induced diarrhea (Table 3). Rigo-Adrover et al. showed that oral infusion of a mixture of short-chain galacto-oligosaccharides, long-chain fructo-oligosaccharides and *Bifidobacterium breve* (M-16V) was highly effective in modulating rotavirus-induced diarrhea for suckling rats [174, 175]. And when the probiotics were replaced by the milk fermented by *Bifidobacterium breve* and *Streptococcus thermophilus*, the incidence and severity of diarrhea were also reduced in suckling rats [176, 177].

**Table 3:** The effect of synbiotics on rotavirus infection in some studies

Synbiotics	Doses and duration	Objects	Rotavirus treatment	Effects of synbiotics	References
A prebiotic mixture (short-chain galacto-oligosaccharides and long-chain fructo-oligosaccharides (9:1)) and *Bifidobacterium breve* (M-16V)	Oral infusion; the prebiotic mixture (0.8 g/100 g body weight, daily); *Bifidobacterium breve* (M-16V, 4.5 × 10^8^ CFU/100 g body weight, daily), 11 or 18 d	3-day-old Lewis rats	On d 5, simian rotavirus (SA-11, 2 × 10^8^ TCID_50_/rat)	This synbiotics improved diarrhea, reduced viral shedding, and enhanced immunity in rotavirus-infected rats	[174]
A prebiotic mixture (short-chain galacto-oligosaccharides and long-chain fructo-oligosaccharides (9:1)) and *Bifidobacterium breve* (M-16V)	Oral infusion; the prebiotic mixture (0.8 g/100 g body weight, daily); *Bifidobacterium breve* (M-16V, 4.5 × 10^8^ CFU/100 g body weight, daily), 12 d	3-day-old Lewis rats	On d 4, simian rotavirus (SA-11, 2 × 10^8^ TCID_50_/rat), and on day 15, mice rotavirus (1.3 × 10^8^ TCID_50_/rat)	This synbiotics alleviated diarrhea, reduced viral shedding after the first infection, and regulated immune responses in rotavirus-infected rats	[175]
A prebiotic mixture (short-chain galacto-oligosaccharides and long-chain fructo-oligosaccharides (9:1)), *Bifidobacterium breve* and *Streptococcus thermophilus* postbiotics, and the combination	Oral infusion; the prebiotic mixture (0.8 g/100 g body weight, daily); the milk fermented by *Bifidobacterium breve* and *Streptococcus thermophilus* (0.92 g/100 g body weight, daily), 15 d	2-day-old Lewis rats	On d 5, simian rotavirus (SA-11, 2 × 10^8^ TCID_50_/rat)	The incidence and severity of diarrhea were significantly reduced by the synbiotics. These administrations regulated the immune function and gut microbiota in rats	[176]
A prebiotic mixture (short-chain galacto-oligosaccharides and long-chain fructo-oligosaccharides (9:1)), and *Bifidobacterium breve* (C50) and *Streptococcus thermophilus* (065) postbiotics	Oral infusion; the prebiotic mixture (0.8 g/100 g body weight, daily); the milk fermented by *Bifidobacterium breve* (C50) and *Streptococcus thermophilus* (065) (3 g/100 g body weight, daily), 19 d	3-day-old Lewis rats	On d 7, simian rotavirus (SA-11, 2 × 10^8^ TCID_50_/rat)	This synbiotics reduced the clinical symptoms of diarrhea (such as severity and incidence), and enhanced the immune response in rats	[177]

No many studies compare the efficiency of different probiotics and/or prebiotics treating and preventing rotavirus infection. Gagnon et al. [97] reported that some Bifidobacteria (such as *Bifidobacterium thermacidophilum* (RBL69, RBL70), *Bifidobacterium longum* (ATCC 15707) and *Bifidobacterium pseudolongum* (ATCC 25526)) were ineffective to rotavirus attachment and infection, but* Bifidobacterium thermacidophilum* (RBL67) could reduce rotavirus infection in mice. Rigo-Adrover et al. compared the efficiency of probiotics, prebiotics and synbiotics on rotavirus-infected rats, and found that the alleviating effect of synbiotics (mixture of short chain galacto-oligosaccharides, long chain fructo-oligosaccharides and *Bifidobacterium breve* (M-16V)) on rotavirus infection was better than that of single probiotics or prebiotics [174, 175].

However, the application of prebiotics and/or probiotics exists on the potential drawbacks or limitations, as following. (1) Individual differences. The composition of gut microbiota is different in each animal. This will lead to the various effects of prebiotics and/or probiotics. Some probiotic strains may not be beneficial, and even have negative effects to hosts [178]. (2) Dosage and usage. The optimal dosage and usage of most prebiotics and probiotics for animals are not yet clear. Different kinds of animals require different doses and usage cycles. Liu et al. [112] found that the low dose of *Lactobacillus acidophilus* (NCFM™) administration did not alleviate rotavirus infection in the gnotobiotic pigs. In addition, the use of prebiotics and probiotics may only be applicable to specific animals or specific health status [179]. (3) Product quality and cost. Nowadays, the quality and effectiveness of many probiotic and probiotic products are unstable for animals in the market. Some products may have good effects, but they is too expensive to use in animal breeding [180].

## Mechanisms of probiotics and/or prebiotics against rotavirus

**Fig. 1:**
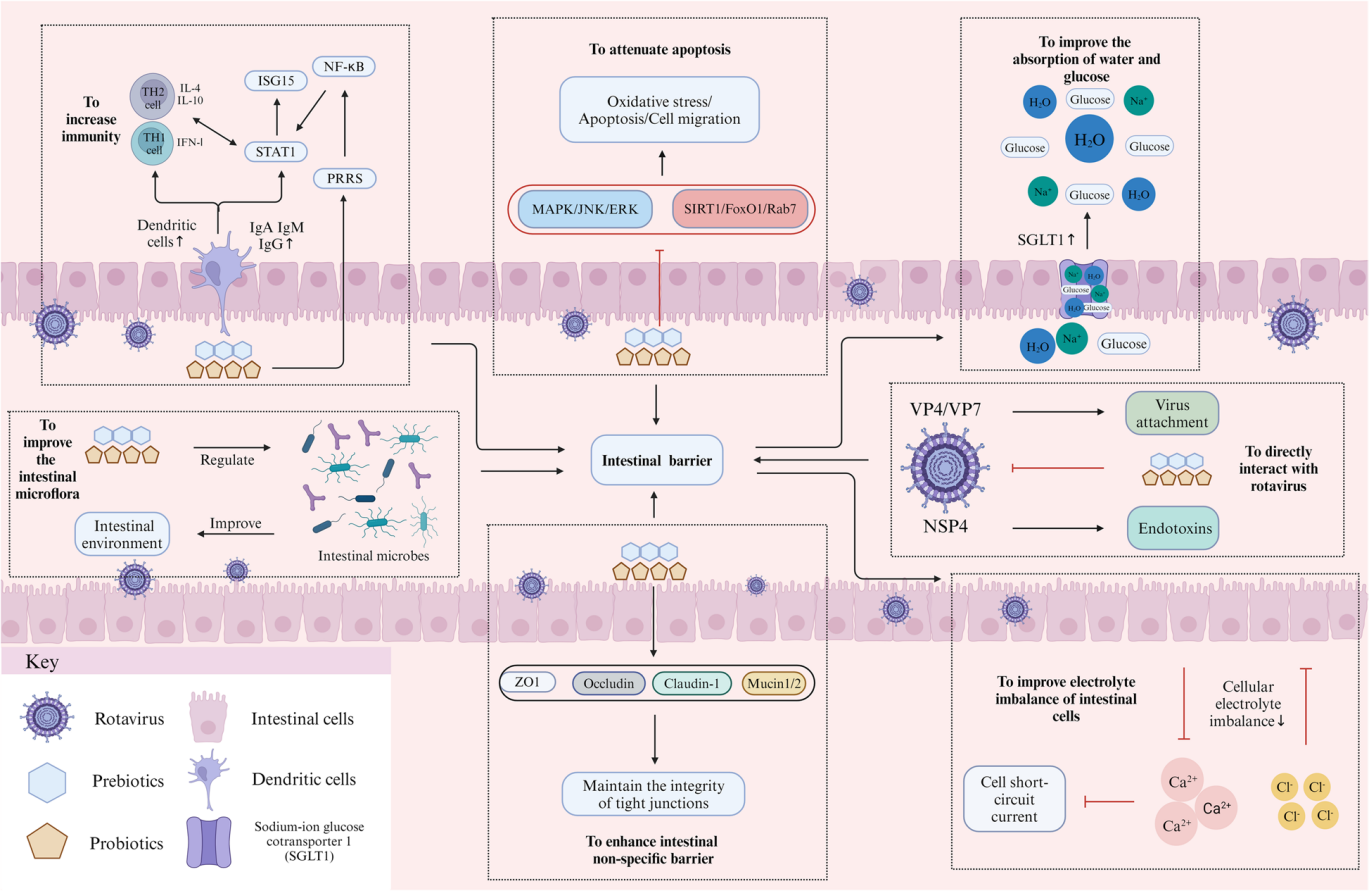
The mechanisms of probiotics and/or prebiotics against rotavirus

Rotavirus is one of the main causes of diarrhea in young animals, especially piglets, worldwide. It can invade hosts (especially intestine), thereby damaging host health and growth. According to the impairment of rotavirus on host (especially intestine) health, many studies analyzed the related indexes, and found that probiotics and/or prebiotics against rotavirus should be involved into several mechanisms, as follows (Fig. 1). (1) To increase immunity. Probiotics and/or prebiotics regulate the activity and function of immune cells (for example dendritic cells), and activate STAT1 through pattern recognition receptors and NF-κB signaling pathways, thereby increasing the production of type I interferon and interleukin, and activating ISG15 and immune response. In addition, probiotics and/or prebiotics can also increase the number of specific antibodies IgG in hosts. (2) To attenuate apoptosis. Probiotics and/or prebiotics might improve intestinal cell apoptosis induced by rotavirus infection through elevating antioxidant capacity and regulating some related signaling pathways (including MAPK/ERK/JNK, SIRT1/FoxO1/Rab7). (3) To enhance intestinal non-specific barrier. Probiotics and/or prebiotics improve the intestinal morphology, and the production of mucins and tight junction proteins. (4) To improve the absorption of water and glucose. Probiotics and/or prebiotics stimulate the mRNA expression and function of sodium/glucose cotransporter 1, thereby improving the water and glucose absorption of rotavirus-infected hosts. (5) To improve the intestinal microflora. Probiotics and/or prebiotics can maintain the balance of gut microbiota, and relieve the negative impact of rotavirus infection on intestinal microflora in hosts. (6) To directly interact with rotavirus. Probiotics and/or prebiotics can bind with VP4 and/or VP7 of rotavirus, thereby reducing the number of rotavirus-infected cells. In addition, probiotics and/or prebiotics can reduce NSP4 levels in the intestine of rotavirus-infected hosts.

### To regulate intestinal microflora

The administration of probiotics and/or prebiotics could decline the negative effects of rotavirus infection on intestinal microflora in hosts. Preidis et al. [77] found that oral administration of *Lactobacillus reuteri* can increase the species richness and phylogenetic diversity of gut microbiota, and alleviate the impairment of rotavirus on gut microbiota in mice. In addition, adding breast milk oligosaccharides in formula can increase the level of *Bacteroidetes* in the colon of newborn piglets infected by rotavirus, thereby alleviating the imbalance of gut microbiota [76]. Our study also found that supplementing the diet with probiotics (i.e., LGG) or prebiotics (including pectic oligosaccharides, lactose, or shiitake polysaccharides) increased the number of *Lactobacillus*, *Bifidobacterium*, and total bacteria in the cecum of rotavirus-infected piglets, and reduced the number of *Escherichia coli* [28, 29, 33, 76]. This can be the main mechanism of probiotics and prebiotics improving host health.

### To improve immunity

The administration of probiotics and/or prebiotics can effectively regulate the immune response of hosts. There are the different roles among different probiotics and prebiotics.

#### Innate immunity and inflammation

The innate immunity and inflammation are the important compounds of immune system of hosts. They mainly contain the activation and effect of immune-related cells, the production and effect of cytokines, and the related pathways. Following the invasion of pathogens, they immediately play a critical role of pathogen clearance in hosts [181]. Probiotics and prebiotics may influence these effects.

1. EcN. Kumar et al. [182] used Gram-positive and Gram-negative probiotics to incubate the rotavirus-infected cells, determined the pathways of innate immunity and inflammation by PCR arrays, and found that EcN activated innate immune (including cell movement of granulocytes, immune response of cells, activation of phagocytes, and activation of macrophages) and inflammatory responses (such as cytokine and chemokine mediated signaling pathway) to neutralize rotavirus infection. In the rotavirus-challenge piglets, oral infusion of EcN increased the function of NK cells and the production of a large number of plasmacyte-like dendritic cells, and reduced the monocytes expressing Toll-like receptor 4 and cytokine (for example IL-6) levels in the spleen, blood, and/or intestine [121–123, 125].
2. *Lactobacillus*, *Lactococcus lactis* and *Ligilactobacillus salivarius*. Oral administration of LGG reduced the percentage of CD11c^+^CD11b^+^CD8a^−^ dendritic cells and increased the percentage of CD11c^+^CD11b^−^CD8a^+^ dendritic cells, thereby regulating immune cells against rotavirus invasion [54]. Liu et al. [108] found that LGG treatment might regulate the expression of Toll-like receptors (TLRs), and some cytokine responses in the rotavirus-infected IPEC-J2 cells. Our study showed that supplementing LGG in diet relieved the effect of rotavirus on IL-2 and IL-4 levels in the jejunum of piglets [33]. Dietary *Lactobacillus reuteri* and *Lactobacillus acidophilus* supplementation could enhanced the response and level of Th1 and Th2 cytokines (i.e., IL-12, IFN-γ, IL-4 and IL-10) in gnotobiotic pigs infected by rotavirus [114]. Oral administration of heat-killed Lactococcus lactis (subsp. Lactis JCM 5805) upregulated the expression levels of anti-viral factors induced by IFNs, such as Isg15, Mx1, Oasl2 and Viperin in the small intestinal epithelial cells of mice [127]. The treatment of Ligilactobacillus salivarius *Ligilactobacillus salivarius* (FFIG35 and FFIG58) stimulated the expression of IFN-β, IFN-λ and antiviral factors in the porcine intestinal epithelial cells infected by rotavirus, which was associated with modulating TLR signaling pathway [130].
3. *Bifidobacterium*. *Bifidobacterium animalis* (subsp. Lactis Bb12) treatment regulated the response of cytokines (such as IL-6, IL-10) via affecting the expression of TLRs and the number of immune cells in the ileal monocytes of piglets [103–105]. Oral administration of *Bifidobacterium longum* (SPM1205 and SPM1206) regulated IFN-mediated innate immunity in rotavirus-infected mice, which was involved into the increase of *IFN-α* and *IFN-β* levels and the mRNA expression of *IFN* receptor, *STAT1* and IFN-induced antiviral effectors (myxovirus resistance A, and 20,50-oligoadenylate synthase) [101].
4. Prebiotics. Supplementing human milk oligosaccharides in formula increased systemic NK cells, memory effector T cells, and major histocompatibility complex II cells in rotavirus-infected pigs [55]. The studies of our lab have shown that the administration of prebiotics (including lentinan, pectic oligosaccharide, or lactose) could improve the concentrations and mRNA expression of cytokines and host defense peptides, and regulate the expression of pattern recognition receptors in the jejunum of rotavirus-challenge piglets [28, 70, 75]. The administration of *Glycyrrhiza uralensis* extract regulated the expression of cytokines via some signaling pathway (such as p38, JNK and NF-κB) in the small intestine and spleen of rotavirus-infected pigs [167]. In the studies of some animal models infected by rotavirus, the administration of prebiotics (such as 2´-fucosyllactose and/or a mixture of short-chain galacto-oligosaccharides/long-chain fructo-oligosaccharides, ziyuglycoside II, *Macleaya cordata* extracts, *Valeriana jatamansi* Jones extracts) could modulate the cytokine expression and generation via some signaling pathways (including pattern recognition receptors, TLR/NF-κB, JAK2/STAT3, PI3K/AKT) [151, 153, 161, 165, 170].

#### Antibody generation

After invading hosts, pathogens are recognized by antigen-presenting cells (such as phagocytes, macrophages, dendritic cells), and then immunocompetent cells are activated [80]. In humoral immunity, plasmacyte will generate the relative antibodies. The antibodies can inhibit pathogen attachment, invasion and proliferation, neutralize pathogens, and boost the pathogen clearance. Probiotics and prebiotics may promote the production of antibodies [183]. In some studies of mouse model, the administration of probiotics (including *Bifidobacterium bifidum*, *Bifidobacterium infantis*, *Bifidobacterium thermophilus*, *Lactobacillus reuteri* and *Lactobacillus gasseri*) increased fecal and serum rotavirus-specific IgA levels in the rotavirus-challenge mice, and even stimulated the humoral specific IgG and IgM responses in the rotavirus-challenge mice [77, 96, 97, 117]. Azevedo et al. [114] and Zhang et al. [115] found that the treatment of *Lactobacillus reuteri* and *Lactobacillus acidophilus* could enhance intestinal IgA-secreting cellular responses, serum IgM titers, and intestinal IgM and IgG titers in rotavirus-infected piglets. Oral gavage of EcN in the rotavirus-challenge gnotobiotic piglets increased the IgA level and response of small intestine [124]. Our studies also found that supplementing probiotics (i.e., LGG) or prebiotics (i.e., lentinan, pectic oligosaccharide, lactose) in diets increased the levels of IgA, IgG or rotavirus-specific antibody in the serum and/or intestine of rotavirus-infected piglets [28, 33, 70, 75]. In addition, many studies reported that probiotics and/or prebiotics administration might enhance the rotavirus-antibody titer and immune response in the hosts with rotavirus vaccine [105–107, 111, 112, 119, 122, 126, 149].

### To maintain intestinal functions

The intestine has the function of digestion, absorption and barrier, which will play a critical for the growth and health of animals and human. The gut barrier encompasses some elements, such as mucosal integrity, and the intercellular junctions between epithelial cells [184]. LGG administration ameliorated morphology, mucin 1 and mucin 2 concentrations, and zonula occludens 1 and occludin expression in the jejunum of rotavirus-infected piglets [33], and increased mucin 3 level in the large intestine digesta of rotavirus-infected gnotobiotic pigs [9]. Oral gavage of *Bifidobacterium bifidum* (G9-1) increased the number of acidic mucin-positive goblet cells, and the mRNA expression of mucin 2, mucin 3, mucin 4, transforming growth factor β1, Trefoil factor 3, occludin, claudin-1 and villin-1 in the small intestine of rotavirus-infected mice [98]. Kumar et al. [182] analyzed the effect of EcN on rotavirus-infected cells through PCR arrays, and found that EcN upregulated intercellular contact and adhesion (especially formation of tight junctions). Our studies also found that prebiotics (i.e., lentinan, pectic oligosaccharide, lactose) administration for rotavirus-infected piglets improved morphology, the levels of mucins, and the expression of tight junction proteins in the jejunum [28, 29, 31]. Altogether, probiotics/prebiotics relieve the damage of rotavirus infection on the host intestinal mucosa by increasing the levels of mucins, tight junction proteins, and some other factors that affect cell growth and repair.

Except for barrier function, the function of digestion and absorption will be impaired by rotavirus infection. However, there are few studies about the effect of probiotics and/or prebiotics on digestion and absorption functions in hosts. Kawahara et al. [98] reported that oral infusion of *Bifidobacterium bifidum* (G9-1) stimulated the mRNA expression of sodium/glucose cotransporter 1 (*SGLT1*), thereby ameliorating water and glucose absorption in the rotavirus-infected mice. Song et al. [159] showed that baicalin administration might modulate the glucose metabolism, which mainly regulate the gluconeogenesis via affecting p-JNK/PDK1/AKT/SIK2 signaling pathway in the mice infected by rotavirus.

The gut functions are closely associated with the survival and activity of intestinal epithelial cells [185]. And probiotics and/or prebiotics might decrease cell apoptosis, and increase cell migration during rotavirus infection, which was due that probiotics and/or prebiotics elevated antioxidant capacity, and regulated some related signaling pathways (including MAPK/ERK/JNK, SIRT1/FoxO1/Rab7) [28, 29, 31, 118, 186–188].

### Some other mechanisms

In the process of probiotics and/or prebiotics against rotavirus infection and rotavirus-induced diarrhea, there are some other ways. (1) To directly interact with external viral proteins. EcN or *Bifidobacterium longum* (SPM1205 and SPM1206) might bind VP4 and/or VP7 of rotavirus, and then reduce the number of virus-infected cells [101]. (2) To reduce NSP4. Our studies showed that the administration of probiotics (i.e., LGG) or prebiotics (i.e., lentinan, pectic oligosaccharide, lactose) decreased the NSP4 level in the intestine of rotavirus-challenge pigs [28, 29, 33, 70]. (3) To decrease Ca^2+^ outflow in cells. Some lactobacilli (i.e., LGG) and their metabolites downregulated the size of short-circuit current and the release of Ca^2+^ in the virus-infected cells [186], and some plant extract (i.e., red wine extract, loureirin C) could inhibit calcium-activated chloride channels in the rotavirus-infected mice [162, 166].

## Conclusion

Rotavirus is one of the main causes of viral diarrhea in piglets worldwide. Probiotics and/or prebiotics have the capacity of promoting the health of pigs. They may, to some extent, prevent and cure rotavirus infection, and attenuate the negative effect of rotavirus infection on health and growth of hosts. In swine production, lactic acid bacteria and bifidobacteria have been widely applicated as the most effective probiotics, while prebiotics are mainly various plant extracts. Their functions are involved into restoring intestinal function, improving digestion and absorption capacity, reducing diarrhea symptoms, regulating gut microbiota, and enhancing immune function. Nowadays, there are many studies on probiotics and/or prebiotics healing and preventing rotavirus infection, which often get the good efficiency. This will supply the effective solution for the prevention of diarrhea (especially rotavirus-induced diarrhea) in the industry of swine breeding.

However, there are many studies needed to be done in future. Firstly, the mechanisms of probiotics and/or prebiotics protecting the hosts against rotavirus infection are not enough. In the second place, there are few studies on the comparation of the efficiency of different probiotics and/or prebiotics treating and preventing rotavirus infection. Last but not the least, during treating rotavirus infection, probiotics and/or prebiotics are mainly used as adjuvant therapy. The related dosages and usage methods should be profoundly researched.

## Data Availability

Not applicable.

## Abbreviations

AMPK: AMP-dependent protein kinase
CAMK2: Calcium/calmodulin-dependent kinase II
EcN: *Escherichia coli* Nissle 1917
ERK: Extracellular regulated protein kinases
GLP-2: Glucagon like peptide-2
IFN: Interferon
Ig: Immunoglobulin
IL: Interleukin
IRF: Interferon regulatory factors
ISG: Interferon-stimulated genes
JNK: JUN N-terminal kinases
LGG: *Lactobacillus rhamnosus* GG
MAPK: Mitogen-activated protein kinases
NF-κB: Nuclear factor-kappa B
Nrf2: Nuclear factor erythroid 2-related factor 2
NSP: Viral non-structural proteins
PRRs: Pattern recognition receptors
SFB: Segmented filamentous bacteria
SGLT1: Sodium/glucose cotransporter 1
TBK1: TANK-binding kinase 1
VP: Virus structure protein
